# Analysis and Application of a 3D Chaotic System with Flexible Offset and Frequency Control

**DOI:** 10.3390/e28030260

**Published:** 2026-02-27

**Authors:** Shuaishuai Shi, Jiangfan Xiong, Licai Liu, Chuanhong Du

**Affiliations:** 1 School of Information Engineering, Guizhou University of Engineering Science, Bijie 551700, China; gues2017@126.com (S.S.); xjf070701@163.com (J.X.); 2 College of Electronics and Information Engineering, Anshun University, Anshun 561000, China; duchuanhong@hrbeu.edu.cn

**Keywords:** chaotic system, offset boosting, frequency regulation, complexity enhancement, synchronization control, data encryption

## Abstract

Signals with flexible control over polarity and frequency provide an essential foundation for reliable and high-speed information transmission. To generate chaotic signals with flexible output characteristics in low-dimensional systems, a novel chaotic system model is proposed by introducing a nonlinear term into the classical Chen chaotic system. Dynamical analysis and MATLAB numerical simulations show that the system is not only highly sensitive to initial conditions but also capable of generating three distinct chaotic attractors. Further simulations confirm that the proposed system demonstrates arbitrary unidirectional and multidirectional offset boosting behaviors, with offset amplitudes in all directions having a wide adjustable range. Furthermore, arbitrary offset constants can effectively control the frequencies of all state variables. This chaotic system, which combines flexible offset control with frequency regulation, is rare in existing research. Additionally, certain parameter ranges in the chaotic regime are relatively narrow. To address this, a method involving control constants to enhance system complexity is proposed, and its effectiveness in increasing system complexity is validated through Lyapunov spectrum and spectral entropy (SE) analysis. Based on the constructed chaotic system, an equivalent circuit model was built using the Multisim 14.0 platform. Experimental results confirm that the system generates chaotic attractors with distinct structures and demonstrates offset boosting behavior in arbitrary directions. Additionally, DSP hardware experiments further validate the physical realizability of the system. To fully exploit the system’s advantages, a synchronization controller was designed for both the drive and response systems, enabling synchronization control of the chaotic system with three offset constants. Based on this, data encryption and transmission experiments were conducted, further establishing the theoretical and experimental foundation for applying the new chaotic system in secure communication.

## 1. Introduction

Chaotic systems typically exhibit high complexity due to their pronounced pseudo-random characteristics and extreme sensitivity to initial conditions. Consequently, they have attracted extensive attention and have been widely applied in various fields, including weak signal detection [1], neural networks [2,3], secure communications [4,5], and control engineering [6,7]. In these application scenarios, chaotic systems with high complexity are of particular significance. The construction of chaotic systems possessing high complexity not only holds substantial theoretical research value but also plays an important role in practical engineering applications. In recent years, the development of novel chaotic system models has gradually become an important research direction in the field of chaotic dynamics. A variety of chaotic systems have been successively proposed, including integer-order chaotic systems [8,9], fractional-order chaotic systems [10,11], discrete chaotic systems [12,13], and conservative chaotic systems [14,15]. These systems have not only enriched the theoretical framework of chaotic dynamics but have also, to some extent, enhanced system complexity, thereby providing a theoretical foundation for applications of chaotic systems in secure communications and related fields. To further increase the complexity of chaotic systems, some studies have focused on high-dimensional chaotic systems, such as five-dimensional (5D) systems [16,17] and 6D systems [18,19]. Although increasing the system dimension can facilitate the generation of complex chaotic behaviors, it also leads to a significant increase in hardware implementation complexity, which consequently limits practical engineering applications. Therefore, compared with high-dimensional systems, the realization of complex chaotic behavior in low-dimensional chaotic systems remains a problem worthy of in-depth investigation. Based on the above considerations, this paper constructs a new 3D chaotic system by introducing nonlinear terms into the classical 3D Chen chaotic system. While maintaining a low system dimension, the proposed system is capable of generating three chaotic attractors with distinct structures and exhibits high sensitivity to initial conditions, thereby providing a new approach for the flexible design and implementation of low-dimensional chaotic systems in engineering applications.

In practical engineering applications, chaotic signals are often required to exhibit unipolar output characteristics. However, chaotic signals typically belong to ultra-wideband low-frequency signals. As a result, polarity conversion usually requires the introduction of large-capacitance components or wideband adders, which leads to increased circuit complexity and higher implementation costs [20]. The team led by Chunbiao Li introduced a constant into the chaotic system and revealed the constant-induced offset boosting behavior [21]. Chaotic systems with offset boosting characteristics are of great significance for chaotic signal modulation and attractor control, as signal polarity conversion can be achieved inherently by the system itself, thereby significantly reducing implementation cost. Consequently, such systems have gradually attracted increasing research interest. Early studies on offset boosting typically relied on additional control functions, such as periodic functions or piecewise functions [22,23]. With further investigations, it was found that in memristor-based chaotic systems, offset regulation of chaotic sequences can be achieved solely by varying initial conditions, a behavior referred to as initial-dependent offset boosting [24]. Some researchers have reported that, in Hopfield neural networks, initial conditions can induce offset boosting behaviors in both chaotic attractors and periodic attractors [25]. Subsequently, multidimensional offset boosting phenomena were reported within a single system, providing greater flexibility for the utilization of chaotic signals [26,27]. To further enhance the controllability of chaotic signals, some studies have proposed chaotic systems capable of simultaneously regulating signal polarity, amplitude, or frequency [20]. In addition, chaotic systems with large-range or even ultra-large-range offset boosting capabilities have also been reported, thereby offering broader design flexibility for engineering applications of chaotic signals [28,29]. On this basis, to further improve the flexibility of chaotic systems, this paper proposes a new 3D chaotic system that enables flexible control of both signal polarity and frequency. The proposed system exhibits arbitrary unidirectional and multidirectional offset boosting behaviors, with all offset constants having wide adjustable ranges. Notably, each offset constant can not only regulate signal polarity but also influence the frequencies of all state variables. Such a high degree of flexible regulation is relatively rare among existing low-dimensional chaotic systems.

The complexity of a chaotic system is commonly used to quantify the degree to which a chaotic sequence approaches a random sequence. A higher complexity value indicates stronger randomness and, consequently, a higher level of security in secure communication applications. At present, the spectral entropy (SE) algorithm has been widely employed for the quantitative analysis of chaotic system complexity [30,31]. Shuaishuai Shi et al. demonstrated that the novel memristor model significantly enhances system complexity [32]. Similarly, Zhang J et al. incorporated memristors into neural network models and confirmed a significant enhancement in system complexity [33]. Although these approaches achieve favorable results in complexity enhancement, they often lead to a substantial increase in system structural complexity, thereby raising the difficulty of hardware implementation.

For the chaotic system constructed in this work, a single control constant is introduced to effectively expand the parameter range of chaotic states and further enhance system complexity. This method features a simple structure, intuitive adjustment, and low implementation cost, providing a practical and engineering-oriented reference for complexity enhancement in chaotic systems.

With the rapid development of information technology, data security has become a critical issue faced by various industries. Among the entire data processing chain, information transmission is particularly vulnerable to attacks due to its openness and broad attack surface, and it is also difficult to protect effectively. Therefore, employing chaotic systems with high complexity for data encryption and secure transmission is of significant practical importance [34,35]. Some studies have enhanced information security strength and encryption speed by developing high-performance encryption algorithms [36]. In addition, encrypted data transmission has been achieved by modulating parameters of chaotic systems [37]. However, because the admissible ranges of system parameters are usually limited, such methods are susceptible to exhaustive attacks. Moreover, parameter modulation often requires adjusting circuit resistances, which is inconvenient in practice and restricts engineering applications. To address these issues, a method for binary data encryption based on the modulation of system offset constants has been proposed [29]. Nevertheless, this approach typically modulates only a single offset quantity, and the offset is directly reflected in the system state variables, which still poses potential security risks. In the chaotic system constructed in this paper, the offset constants along all three directions have wide adjustable ranges. To fully exploit this advantage, a data encryption method based on the modulation of three offset constants is proposed, in which the employed offsets are not directly manifested in the system state variables. As a result, the encryption security is effectively enhanced, laying a foundation for the application of chaotic systems in secure communication.

At present, various improved chaotic systems have been reported in the literature. Compared with existing systems, the proposed system exhibits certain distinctive advantages in terms of structural characteristics and controllability. A detailed comparison is presented in Table 1.

The organization of this paper is as follows. Section 2 introduces the proposed chaotic system model and provides a brief analysis of its fundamental properties. Section 3 investigates the dynamical characteristics of the proposed system, including sensitivity to initial conditions, dynamical behaviors induced by parameter variations, offset boosting effects caused by control constants, and enhancement of system complexity. Section 4 verifies the physical realizability of the proposed system through Multisim simulations and Digital Signal Processor (DSP) hardware experiments. In Section 5, a synchronization controller is designed to achieve system synchronization and secure data encryption transmission. Finally, Section 6 concludes the paper.

## 2. New System Model and Dynamical Behavior Analysis

### 2.1. Construction of the New System Model

**Figure 1:**
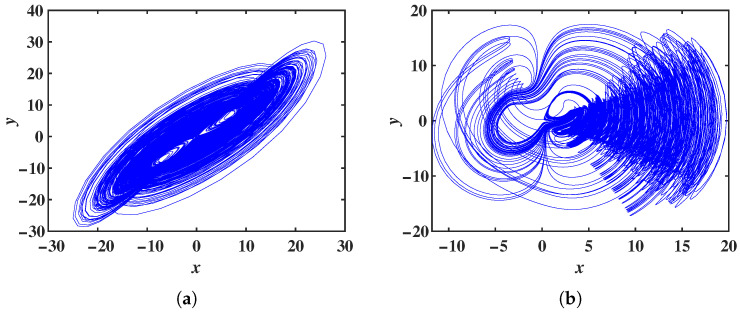
Phase portraits of the two systems for comparison: (**a**) System (1) *x*-*y* phase plane diagram. (**b**) System (2) *x*-*y* phase plane diagram.

The standard form of the Chen chaotic system is a set of 3D autonomous differential equations, as shown in Equation (1) [39]. When parameters a=35, b=3, and c=28, the system exhibits chaotic behavior, with the attractor phase diagram depicted in Figure 1a.

(1) $$\left\{\begin{array}{c} \dot{x}=a\left(y-x\right)\\ \dot{y}=\left(c-a\right)x-xz+cy\\ \dot{z}=xy-bz \end{array}\right.$$

By introducing a cross-product term, a magnitude function, and a hyperbolic tangent function into the first equation of the Chen system, a new chaotic system is established through parameter rescaling. The mathematical model of this new chaotic system is given by Equation (2).

(2) $$\left\{\begin{array}{c} \dot{x}=y-x+yz+m\left|z\right|-n\tanh\left(z\right)\\ \dot{y}=ax+b\left(y-xz\right)\\ \dot{z}=xy-cz \end{array}\right.$$

where *a*, *b*, *c*, *m*, and *n* are system parameters. With initial conditions (1, 1, 1) and parameters a = 0.6, b = 2, c = 3, m = 2, n = 6, the ODE45 solver in MATLAB 2019a simulates the system’s x − y phase diagram shown in Figure 1b. Figure 1b reveals that system (2) exhibits attractors with entirely distinct structures and possesses a high degree of complexity.

### 2.2. Dissipativity and Stability Analysis of Equilibrium Points

(1) Dissipativity analysis

For system (2), the dissipativity can be determined by calculating the divergence of the system, which is given by

(3) $$\nabla V=\frac{\partial \dot{x}}{\partial x}+\frac{\partial \dot{y}}{\partial y}+\frac{\partial \dot{z}}{\partial z}=-1+b-c$$

The dissipativity of the system is determined solely by parameters *b* and *c* and is independent of the parameter *a* or the introduced nonlinear term. Dissipativity implies that the phase-space volume contracts with time, and the system trajectories eventually converge to a low-dimensional attractor. A negative divergence is one of the necessary conditions for the existence of chaotic attractors. For the parameter values a=0.6, b=2, and c=3, the divergence of the system is ∇V=−2<0, indicating that the system is dissipative. Consequently, the phase-space volume decays exponentially at a rate of $e^{-2}$.

(2) Equilibrium point and stability analysis

By setting the right-hand side of system (2) to zero, the equilibrium equations are obtained as

(4) $$\left\{\begin{array}{c} y-x+yz+m\left|z\right|-n\tanh\left(z\right)=0\\ ax+b\left(y-xz\right)=0\\ xy-cz=0 \end{array}\right.$$

Under the parameter settings given in Section 2.1, the equilibrium point of the system can be obtained as $S_{0}=\left(0,\;0,\;0\right)$. The Jacobian matrix corresponding to system (2) is given by

(5) $$J=\left[\begin{array}{ccc} -1 & 1+z & y+m\mathrm{sign}\left(z\right)-n\left(1-\tanh^{2}\left(z\right)\right)\\ a-bz & b & -bx\\ y & x & -c \end{array}\right]$$

The Jacobian matrix of system (2) evaluated at the equilibrium point $S_{0}=\left(0,\;0,\;0\right)$ is

(6) $$J_{0}=\left[\begin{array}{ccc} -1 & 1 & -n\\ a & b & 0\\ 0 & 0 & -c \end{array}\right]=\left[\begin{array}{ccc} -1 & 1 & -6\\ 0.6 & 2 & 0\\ 0 & 0 & -3 \end{array}\right]$$

The characteristic equation of system (2) at $S_{0}=\left(0,\;0,\;0\right)$ can be obtained as

(7) $$\left|\lambda E-J_{0}\right|=\lambda^{3}+2\lambda^{2}-\frac{28}{5}\lambda -\frac{39}{5}$$

According to the Routh–Hurwitz criterion, the equilibrium point $S_{0}=\left(0,\;0,\;0\right)$ is unstable, and the corresponding eigenvalues are $\lambda_{1}=-3,\;\lambda_{2}=-1.1882,\;\lambda_{3}=2.1882$. Based on these eigenvalues, the equilibrium point is identified as an unstable saddle point [40].

### 2.3. Lyapunov Exponents and Kaplan–Yorke Dimension

Lyapunov exponents are important criteria for determining whether a system exhibits chaotic behavior. For system (2), when the parameters and initial conditions are fixed, the three Lyapunov exponents are $LE_{1}=0.465$, $LE_{2}=0$ and $LE_{3}=-2.467$. Since the maximum Lyapunov exponent of the system is greater than zero and the sum of the Lyapunov exponents is less than zero, the system exhibits chaotic behavior. According to the Kaplan–Yorke dimension formula $D_{KY}=j+\frac{\sum_{i=1}^{j}LE_{i}}{\left|\lambda_{j+1}\right|}$, the value of $D_{KY}=2.1885$ for system (2) can be obtained, indicating that the system possesses a fractal dimension. Therefore, the system is capable of generating a strange attractor.

## 3. Dynamical Behavior Analysis

### 3.1. Sensitivity to Initial Conditions

**Figure 2:**
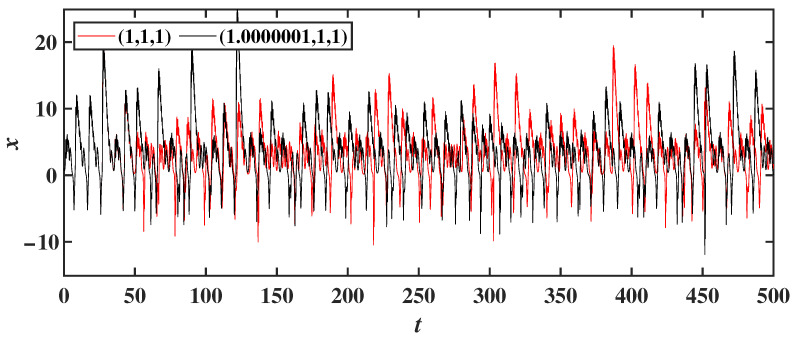
Time-domain trajectories of the variable *x* for initial conditions (1, 1, 1) and (1.0000001, 1, 1).

For system (2), the parameters are chosen as a=0.6, b=2, c=3, m=2 and n=6, with initial conditions (x(0), y(0), z(0)). The values of y(0)=1 and z(0)=1 are fixed, while x(0) is set to 1 and 1.0000001, respectively. The corresponding time-domain trajectories are shown in Figure 2. As illustrated in Figure 2, a slight change in the initial value of x(0) leads to significantly different trajectories after 50 s, demonstrating the extreme sensitivity of system (2) to initial conditions.

### 3.2. Dynamical Behavior Analysis with Respect to the Parameter m

**Figure 3:**
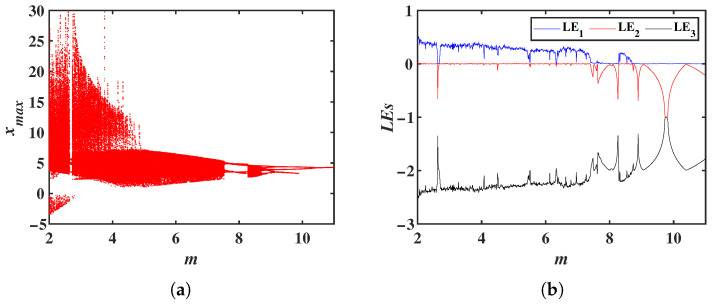
Bifurcation diagram and LEs of system (2) with respect to the parameter *m*: (**a**) Bifurcation diagram. (**b**) LEs.

**Figure 4:**
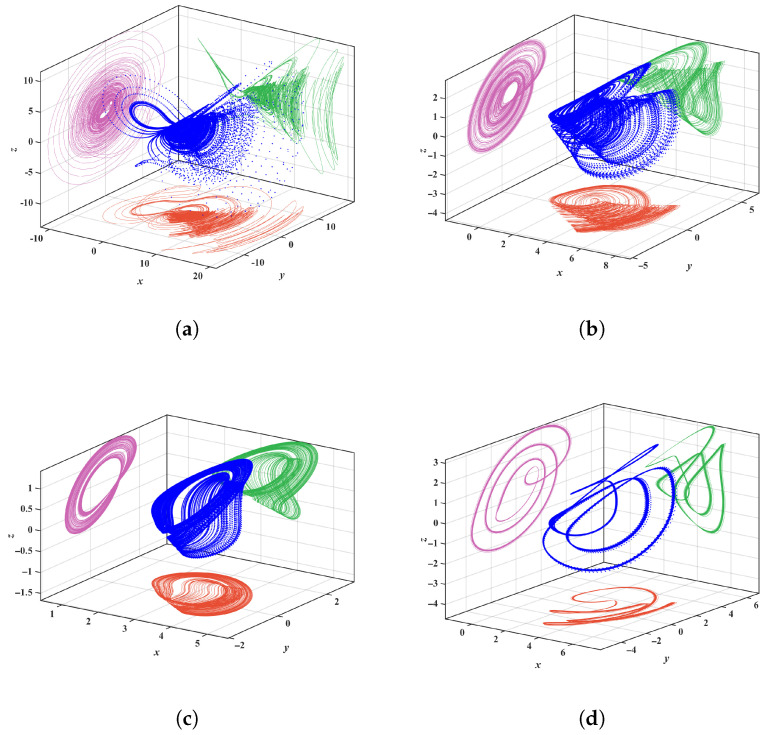
3D phase diagrams of System (2) for different values of *m*: (**a**) m=2.5. (**b**) m=5. (**c**) m=8.5. (**d**) m=2.7.

The system parameters are fixed as a=0.6, b=2, c=3 and n=6, with initial conditions (1, 1, 1). The parameter *m* is varied within the range [2, 11]. Using MATLAB simulations, the Lyapunov exponent spectrum (LEs) and bifurcation diagram of system Equation (2) with respect to are obtained, as shown in Figure 3. From the bifurcation diagram and LEs, it can be observed that within the range m∈[2, 11], system (2) exhibits chaotic behavior for most values in m∈[2, 7.52] and m∈[8.28, 8.72], with narrow periodic windows appearing in between, and the remaining parameter ranges corresponding to periodic behavior. Specifically, for m=2.5, 5, 8.5, and 2.7, the corresponding system attractors are shown in Figure 4. It can be seen that completely different attractor structures emerge for different values of *m*.

### 3.3. Offset Boosting Behavior Induced by Constants

Offset boosting facilitates the generation of unipolar signals, which is beneficial for signal processing and engineering applications. This section focuses on the offset boosting behavior induced by a single constant as well as multiple constants.

#### 3.3.1. Unidirectional Offset Boosting Behavior

To investigate the unidirectional offset boosting behavior induced by a single constant in system (2), all the *x* variables are introduced with an offset constant $p_{1}$, transforming system (2) into system (8).

(8) $$\left\{\begin{array}{c} \dot{x}=y-\left(x+p_{1}\right)+yz+m\left|z\right|-n\tanh\left(z\right)\\ \dot{y}=a\left(x+p_{1}\right)+b\left(y-\left(x+p_{1}\right)z\right)\\ \dot{z}=\left(x+p_{1}\right)y-cz \end{array}\right.$$

**Figure 5:**
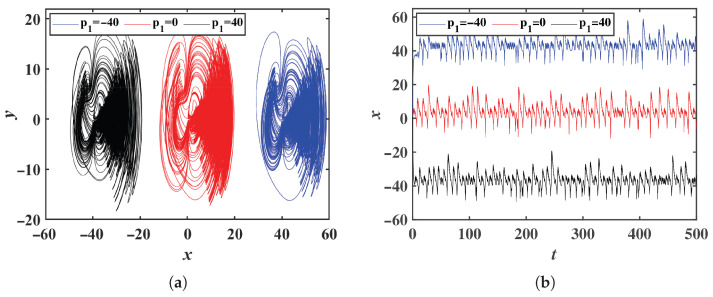
Offset boosting of the variable *x*: (**a**) Phase portraits in the *x*-*y* plane. (**b**) Time-domain waveform of the variable *x*.

The system parameters are the same as those in Section 2.1. When $p_{1}$ is set to −40, 0, and 40, the phase portraits in the *x*-*y* plane and the corresponding time-domain waveforms of system (8) are shown in Figure 5. For $p_{1}=0$, the signal *x* exhibits a bipolar behavior. When $p_{1}>0$, the attractor in the *x*-*y* plane shifts along the negative *x*-axis, converting the bipolar signal *x* into a unipolar signal with negative polarity. Conversely, when $p_{1}<0$, the attractor in the *x*-*y* plane shifts along the positive *x*-axis, resulting in a unipolar signal *x* with positive polarity. Therefore, by introducing the offset constant $p_{1}$ into system (2), the bipolar signal can be transformed into a unipolar signal of arbitrary polarity.

**Figure 6:**
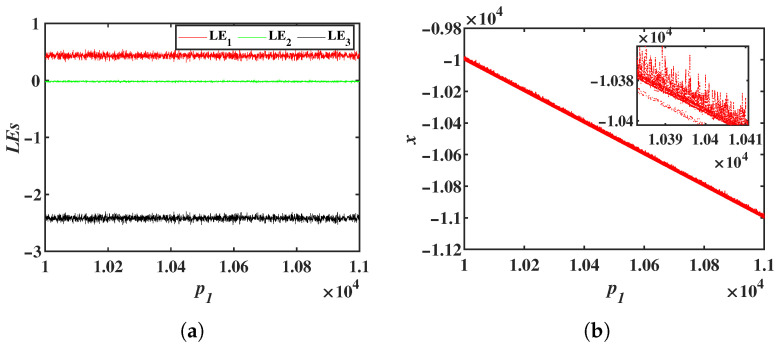
LEs and the bifurcation diagrams of the system (8) as a function of $p_{1}$: (**a**) LEs. (**b**) The bifurcation diagrams.

Simulation results indicate that the offset constant for the variable *x* can span a very wide range. Due to simulation time constraints, $p_{1}\in \left[10,000,\;11,000\right]$ was selected. The LEs and bifurcation diagrams of system (8) as $p_{1}$ varies are presented in Figure 6. From both the LEs and the bifurcation diagrams, it can be observed that as $p_{1}$ increases, the system maintains a chaotic state. From the Lyapunov exponent spectra and bifurcation diagrams, it can be observed that as the offset constant $p_{1}$ increases, the system remains in a chaotic state. The bifurcation diagram further shows that the mean value of variable *x* decreases linearly with increasing $p_{1}$. By selecting different extreme values of $p_{1}$, the LEs and phase portraits of system (8) are shown in Table 2. These results confirm that system (8) exhibits a large-range offset boosting behavior along the *x* direction. Due to limitations in simulation time and computer memory, the upper and lower bounds of the offset constant $p_{1}$ were not identified. In engineering applications, the system can provide chaotic signals with an extremely wide offset range. Furthermore, the offset constant $p_{1}$ also regulates the frequencies of all variables in system (8), with its effect on the frequencies of *y* and *z* shown in Figure 8a,b. To investigate the effect of the offset constant on signal frequency modulation in more detail, the peak frequencies of the variables *y* and *z* with respect to the offset constant $p_{1}$ were plotted, as shown in Figure 8c,d. Chaotic sequences under different offset values were generated using the ODE45 algorithm, and their power spectra were obtained via Fast Fourier Transform (FFT). In the single-sided power spectrum, the frequency corresponding to the maximum amplitude was identified as the dominant frequency.

**Figure 7:**
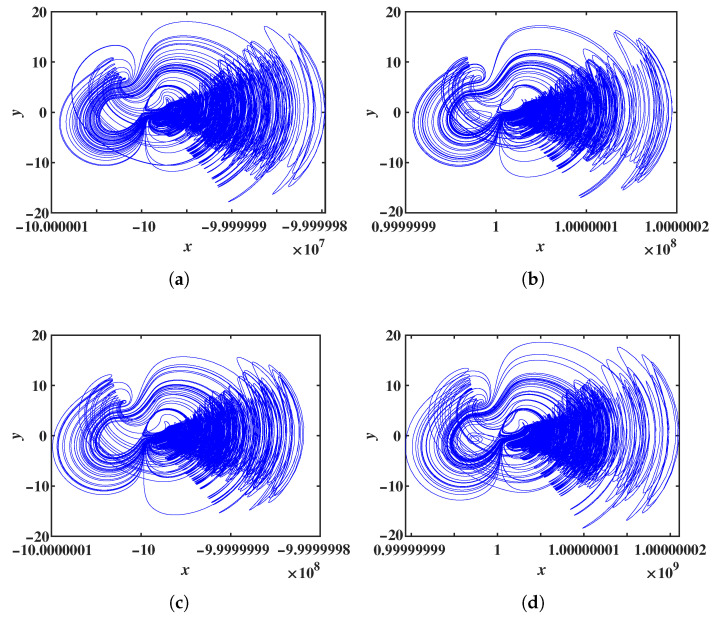
Phase portraits of system (8) in the *x*-*y* plane under the maximum offset $p_{1}$: (**a**) $p_{1}=1\times 10^{8}$. (**b**) $p_{1}=-1\times 10^{8}$. (**c**) $p_{1}=1\times 10^{9}$. (**d**) $p_{1}=-1\times 10^{9}$.

By introducing the offset constant $p_{2}$ to all the *y* variables in system (2), system (2) is transformed into system (9). Similarly, by introducing the offset constant $p_{3}$ to all the *z* variables in system (2), system (2) becomes system (10). The system parameters are the same as those in Section 2.1. When $p_{2}$ takes the values −40, 0 and 40 in system (9), the phase portraits in the *x*-*y* plane are shown in Figure 9a. When $p_{3}$ takes the values −40, 0 and 40 in system (10), the phase portrait in the *x*-*z* plane is shown in Figure 9b. Simulation results indicate that system (9) exhibits offset boosting behavior along the *y* direction, while system (10) shows offset boosting along the *z* direction. Additionally, the simulation confirms that both the offset constants $p_{2}$ and $p_{3}$ can span a wide range of values. Furthermore, a single offset constant regulates the frequencies of all system variables, which is a relatively rare feature for a system with flexible polarity and frequency control.

(9) $$\left\{\begin{array}{c} \dot{x}=y+p_{2}-x+\left(y+p_{2}\right)z+m\left|z\right|-n\tanh\left(z\right)\\ \dot{y}=ax+b\left(\left(y+p_{2}\right)-xz\right)\\ \dot{z}=x\left(y+p_{2}\right)-cz \end{array}\right.$$

(10) $$\left\{\begin{array}{c} \dot{x}=y-x+y\left(z+p_{3}\right)+m\left|z+p_{3}\right|-n\tanh\left(z+p_{3}\right)\\ \dot{y}=ax+b\left(y-x\left(z+p_{3}\right)\right)\\ \dot{z}=xy-c\left(z+p_{3}\right) \end{array}\right.$$

**Figure 8:**
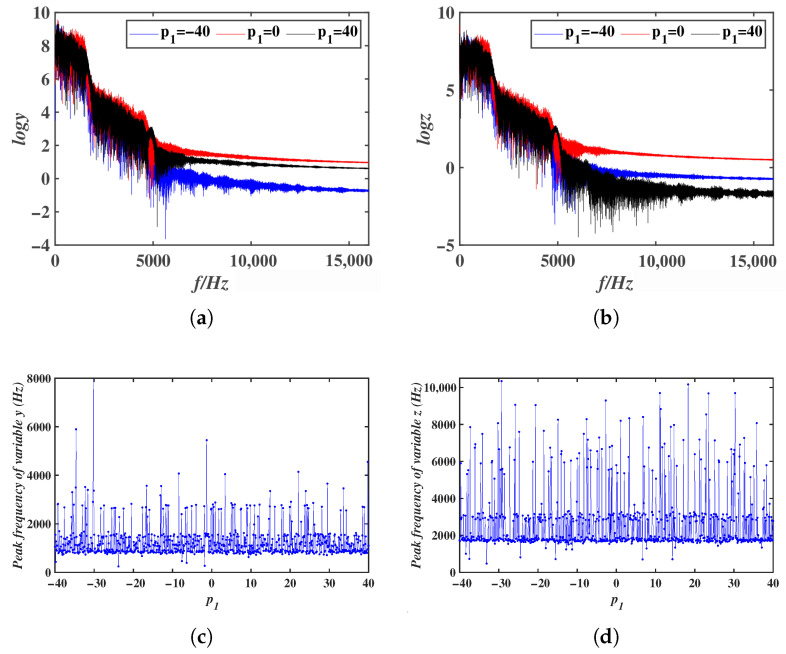
Frequency and peak frequency of system variables under different offset constants $p_{1}$: (**a**) The variable *y*. (**b**) The variable *z*. (**c**) Peak frequency of *y*. (**d**) Peak frequency of *z*.

**Figure 9:**
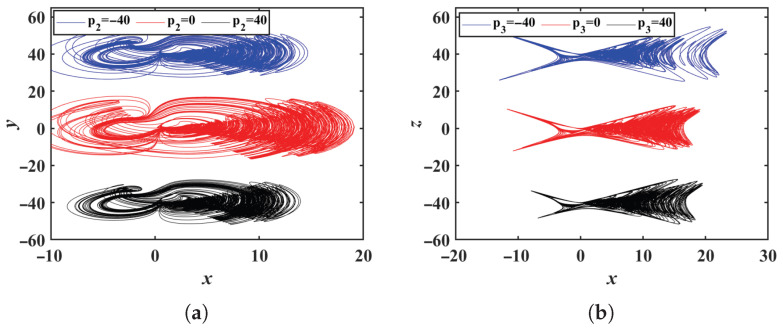
Phase portraits in the *x*-*y* plane with offset boosting: (**a**) In the *x*-*y* plane. (**b**) In the *x*-*z* plane.

#### 3.3.2. Multidirectional Offset Boosting Behavior

By introducing offset constants to multiple variables in system (2), system (2) is transformed into system (11), enabling both 2D and 3D offset boosting behavior. At this stage, the offset constants across multiple directions possess a wide range of values, which will not be reiterated in this section.

(11) $$\left\{\begin{array}{c} \dot{x}=y+p_{2}-\left(x+p_{1}\right)+\left(y+p_{2}\right)\left(z+p_{3}\right)+m\left|z+p_{3}\right|-n\tanh\left(z+p_{3}\right)\\ \dot{y}=a\left(x+p_{1}\right)+b\left(\left(y+p_{2}\right)-\left(x+p_{1}\right)\left(z+p_{3}\right)\right)\\ \dot{z}=\left(x+p_{1}\right)\left(y+p_{2}\right)-c\left(z+p_{3}\right) \end{array}\right.$$

**Figure 10:**
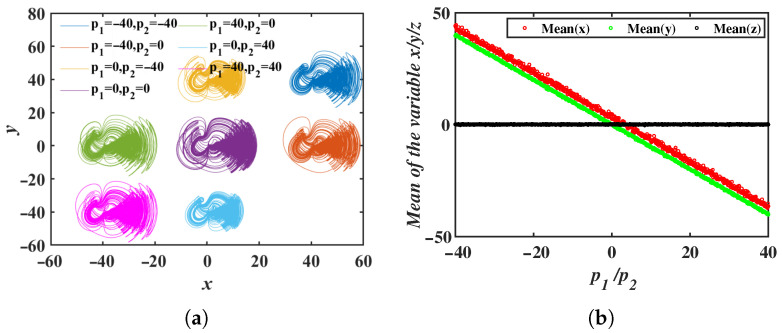
2D offset boosting behavior induced by the offset constants $p_{1}$ and $p_{2}$: (**a**) Phase portraits in the *x*-*y* plane. (**b**) Mean values of the system variables.

When the offset constant $p_{3}=0$, and the offset constants $p_{1}$ and $p_{2}$ are varied, the resulting 2D offset phase portrait is shown in Figure 10a, where the attractor moves within the *x*-*y* plane. Simulation results in Figure 10b indicate that the mean values of variables *x* and *y* decrease as the offset constants increase, while the mean value of *z* remains unchanged. The simulation also confirms that the 2D offset boosting behavior simultaneously occurs in the x−z and y−z directions.

**Figure 11:**
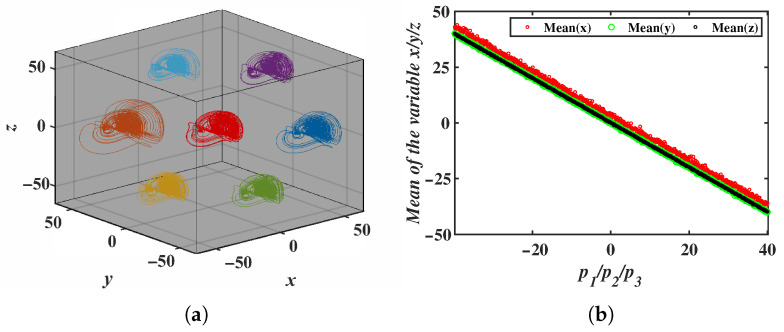
3D offset boosting behavior induced by the offset constants $p_{1}$, $p_{2}$ and $p_{3}$: (**a**) Phase portraits in the *x*-*y*-*z* plane. (**b**) Mean values of the system variables.

The 3D offset boosting behavior for the selected offset constants $\left(p_{1},p_{2},p_{3}\right)=\left(0,\;0,\;0\right),$(−40, 40, 0), (40, −40, 0), (40, 0, 40), (−40, 0, −40), (0, 40, 40), (0, −40, −40) is shown in Figure 11a. The phase portrait illustrates the movement of the attractor in the x−y−z 3D space. In Figure 11b, the mean values of the *x*, *y* and *z* variables change linearly as the offset constants $p_{1}$, $p_{2}$ and $p_{3}$ vary.

The results in this section demonstrate that the proposed 3D chaotic system exhibits offset boosting behavior in any single direction as well as in multiple directions, with a wide range of possible values for the three offset constants. Each offset constant is capable of regulating the frequencies of all system variables. This flexible offset control and frequency regulation provide significant advantages for the engineering applications of chaotic signals.

### 3.4. Complexity Enhancement

In secure communication, chaotic systems are concerned not only with the randomness of the sequences but also with the size of the key space [36]. Under chaotic conditions, a narrow parameter range not only reduces system complexity but also narrows the key space, which is unfavorable for the application of the system in secure communication. Simulation results show that, with other parameters fixed, the parameter *a* in system (2) induces chaotic behavior only within the range a∈[0.2, 1.4]. To expand the range of parameters that lead to the chaotic state and enhance the system’s complexity, a constant is added to system (2). This not only increases the range of parameters that cause the system to enter the chaotic state but also enhances the system’s complexity. For system (2), after adding a constant *p* to the third equation, system (2) is transformed into system (12).

(12) $$\left\{\begin{array}{c} \dot{x}=y-x+yz+m\left|z\right|-n\tanh\left(z\right)\\ \dot{y}=ax+b\left(y-xz\right)\\ \dot{z}=xy-cz+p \end{array}\right.$$

**Figure 12:**
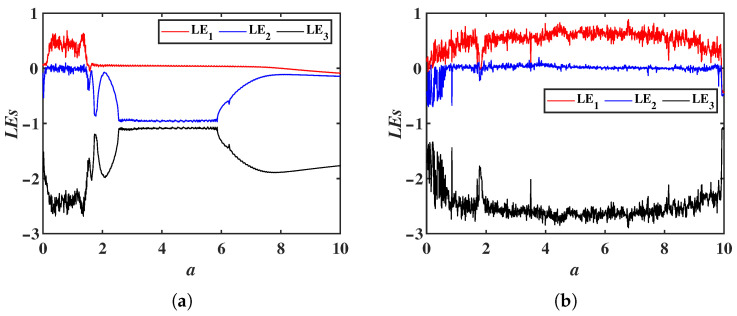
LEs of the system (12) as a function of the parameter *a*: (**a**) p=0. (**b**) p=15.

**Figure 13:**
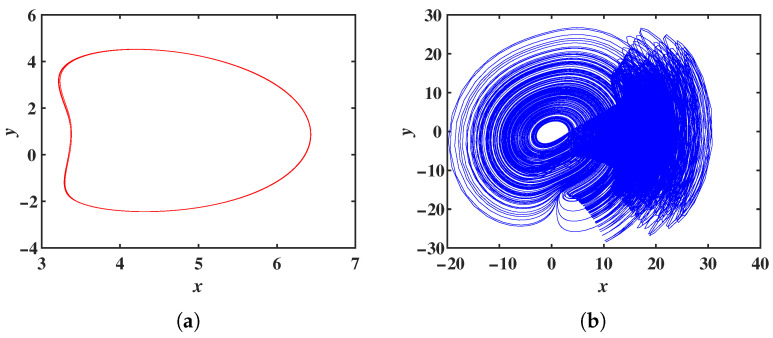
x−y phase portraits of system (12) for different values of *p*: (**a**) p=0. (**b**) p=15.

With fixed parameters b=2, c=3, m=2 and n=6, and initial values set to (1, 1, 1), the parameter *a* is chosen to vary within the range a∈[0, 10]. When the constant p=0, system (12) reduces to system (2), and the LEs as a function of *a* is shown in Figure 12a. For p=15, the LEs of system (12) as a function of *a* is shown in Figure 12b. Comparing these two figures, it can be observed that the chaotic range of system (2) is extended under the influence of the constant *p*. For the parameters a=3, b=2, c=3, m=2 and n=6, when p=0, the phase portrait in the *x*-*y* plane is shown in Figure 13a; when p=15, the phase portrait in the *x*-*y* plane is shown in Figure 13b. The constant *p* also extends the chaotic ranges of the system parameters *c* and *m*.

**Figure 14:**
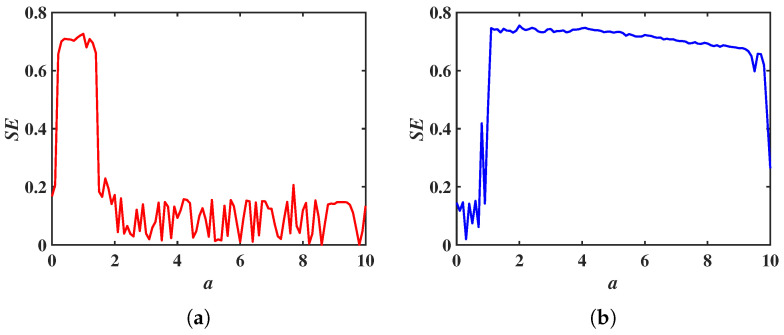
SE of system (12) as a function of the parameter *a*: (**a**) p=0. (**b**) p=15.

To further demonstrate the role of the constant *p* in enhancing system complexity, the SE algorithm is used to compare the complexities of system (2) and system (12). With the parameters fixed at b=2,c=3,m=2,n=6, and the initial values set to (1, 1, 1), the variation range of parameter a is selected as a∈[0, 10]. When the constant p=0, system (12) reduces to system (2). The SE of the system as parameter *a* varies is shown in Figure 14a. Setting the constant p=15, the SE of system (12) as parameter *a* varies is shown in Figure 14b. By comparing Figure 14, it can be observed that the complexity of system (2) is enhanced under the influence of the constant *p*.

To highlight the advantages of the proposed method, the SE values of comparable systems were analyzed and compared, as summarized in Table 3.

## 4. Multisim Circuit Simulation and DSP Hardware Implementation

### 4.1. Multisim Circuit Simulation

To verify the physical feasibility of the new system, the constructed chaotic system (2) is simulated using Multisim 14. Considering the ±15 V power supply limitation of the general-purpose operational amplifier LM358 and the ±10 V linear operating range of the AD633 analog multiplier, normalization of the system parameters is required. By introducing the coordinate transformations $x^{\prime }=10x$, $y^{\prime }=10y$, $z^{\prime }=10z$, the state variables are scaled by a factor of 10. Let $x^{\prime }=u_{x}$, $y^{\prime }=u_{y}$ and $z^{\prime }=u_{z}$, and the adjusted equations are

(13) $$\left\{\begin{array}{c} {\dot{u}}_{x}=u_{y}-u_{x}+10u_{y}u_{z}+m\left|u_{z}\right|-\frac{n}{10}\tanh\left(10u_{z}\right)\\ {\dot{u}}_{y}=au_{x}+b\left(u_{y}-10u_{x}u_{z}\right)\\ {\dot{u}}_{z}=10u_{x}u_{y}-cu_{z} \end{array}\right.$$

**Figure 15:**
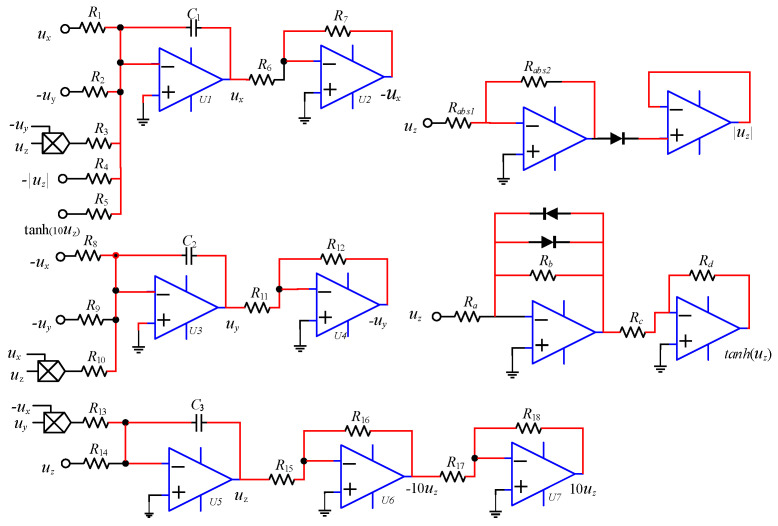
Circuit schematic of the system (13).

The circuit diagram based on Equation (13) is shown in Figure 15, and the circuit equations are given by Equation (14).

(14) $$\left\{\begin{array}{c} {\dot{u}}_{x}=\frac{u_{y}}{C_{1}R_{2}}-\frac{u_{x}}{C_{1}R_{1}}+\frac{g_{1}u_{y}u_{z}}{C_{1}R_{3}}+\frac{\left|u_{z}\right|}{C_{1}R_{4}}-\frac{\tanh(10u_{z})}{C_{1}R_{5}}\\ {\dot{u}}_{y}=\frac{u_{x}}{C_{2}R_{8}}+\frac{u_{y}}{C_{2}R_{9}}-\frac{g_{2}u_{x}u_{z}}{C_{2}R_{10}}\\ {\dot{u}}_{z}=\frac{g_{3}u_{x}u_{y}}{C_{3}R_{13}}-\frac{u_{z}}{C_{3}R_{14}} \end{array}\right.$$

**Figure 16:**
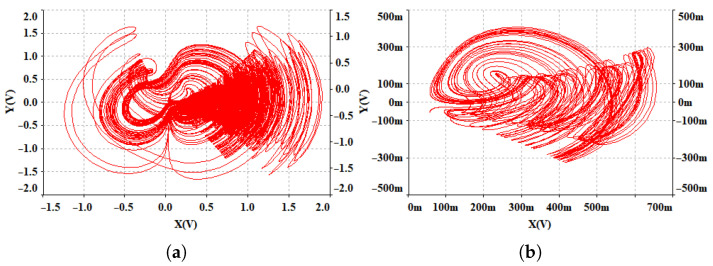
Circuit simulation phase portrait in the *x*-*y* plane: (**a**) m=2.5. (**b**) m=5.

The gains $g_{1}$, $g_{2}$ and $g_{3}$ represent the multipliers. In the absolute value circuit, the resistors are $R_{abs1}=R_{abs2}=100\;k\Omega$, and in the hyperbolic tangent function circuit, the resistors are $R_{a}=900\;\Omega$, $R_{b}=500\;\Omega$, $R_{c}=1\;k\Omega$ and $R_{d}=1.7\;k\Omega$. Let $g_{1}=g_{2}=g_{3}=1$, and the capacitors are $C_{1}=C_{2}=C_{3}=10\;\mu F$. Based on the parameters a=0.6, b=2, c=3 and n=6 from system (13), the following resistor values are calculated: $R_{1}=R_{2}=100\;k\Omega$, $R_{3}=R_{6}=R_{7}=R_{11}=R_{12}=R_{13}=R_{16}=R_{17}=R_{18}=10\;k\Omega$, $R_{5}=R_{8}=166.67\;k\Omega$, $R_{9}=50\;k\Omega$, $R_{10}=5\;k\Omega$, $R_{14}=33.33\;k\Omega$ and $R_{15}=1\;k\Omega$. When m=2.5, the corresponding resistor $R_{4}=40\;k\Omega$, and the circuit simulation x−y phase portrait is shown in Figure 16a. When m=5, the corresponding resistor $R_{4}=20\;k\Omega$, and the circuit simulation phase portrait is shown in Figure 16b. Comparing Figure 4a,b, it can be observed that the Multisim circuit simulation results are in good agreement with the Matlab numerical simulations, confirming that the newly constructed chaotic system exhibits multiple structural attractors and is physically realizable.

**Figure 17:**
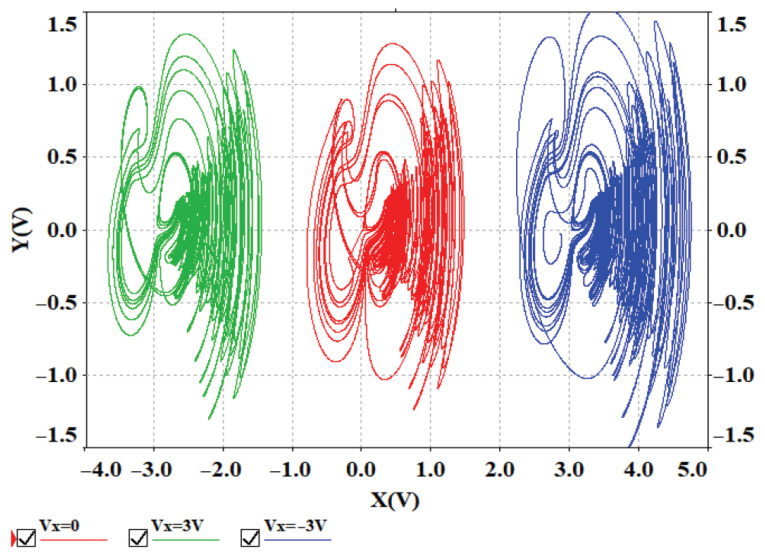
Offset boosting behavior caused by the different voltage constant $V_{x}$ simulated in Multisim.

To verify the offset boosting behavior induced by a single constant, a voltage constant $V_{x}$ is introduced into $u_{x}$ of system (13), transforming Equation (13) into Equation (15). When the voltage constant $V_{x}$ is set to 0 V, 3 V, and −3 V, the phase portraits are shown in Figure 17, which are consistent with the simulation results in Figure 5a, confirming that the offset boosting in the *x* direction is physically realizable. Circuit simulations further confirm that the offset boosting in the *y* and *z* directions is also physically realizable.

(15) $$\left\{\begin{array}{c} {\dot{u}}_{x}=u_{y}-\left(u_{x}+V_{x}\right)+10u_{y}u_{z}+m\left|u_{z}\right|-\frac{n}{10}\tanh\left(10u_{z}\right)\\ {\dot{u}}_{y}=a\left(u_{x}+V_{x}\right)+b\left(u_{y}-10\left(u_{x}+V_{x}\right)u_{z}\right)\\ {\dot{u}}_{z}=10\left(u_{x}+V_{x}\right)u_{y}-cu_{z} \end{array}\right.$$

**Figure 18:**
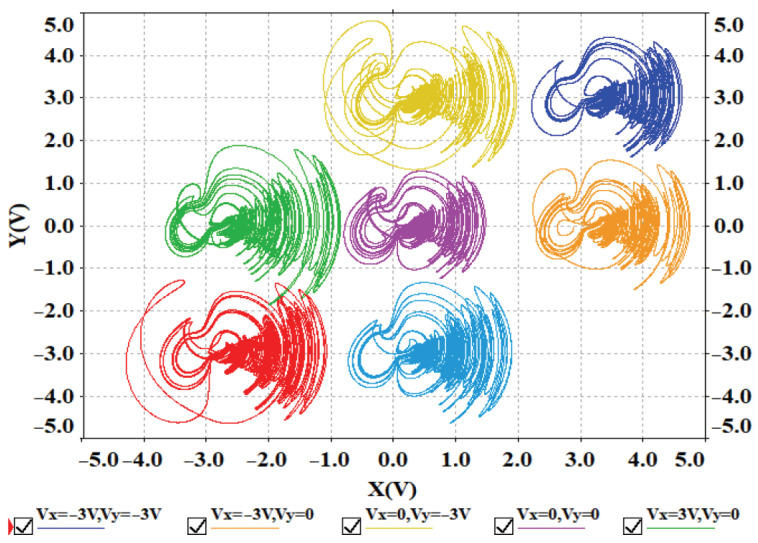
Offset boosting caused by the voltage constant $V_{x}$ and $V_{y}$ simulated in Multisim.

To verify the offset boosting behavior induced by multiple constants, voltage constants $V_{x}$ and $V_{y}$ are introduced to $u_{x}$ and $u_{x}$ in system (13), respectively. The x−y phase portraits for different values of voltage constants $V_{x}$ and $V_{y}$ are shown in Figure 18. The phase portraits of the circuit simulation are in good agreement with the Matlab simulation results in Figure 10a, confirming the presence of offset boosting in both the *x* and *y* directions. Circuit simulations further confirm that offset boosting occurs simultaneously in the *x* and *z* directions, the *y* and *z* directions, as well as in all three directions, *x*, *y* and *z*.

### 4.2. DSP Hardware Implementation

**Figure 19:**
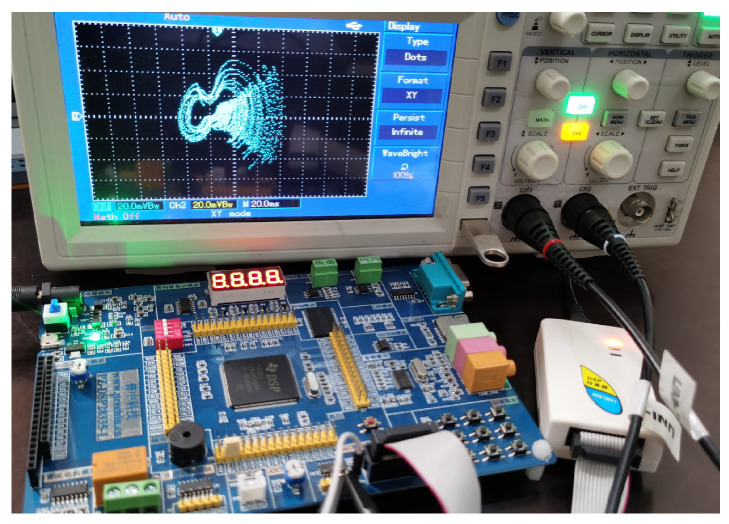
Hardware implementation platform of the DSP-based system with data conversion and signal display.

**Figure 20:**
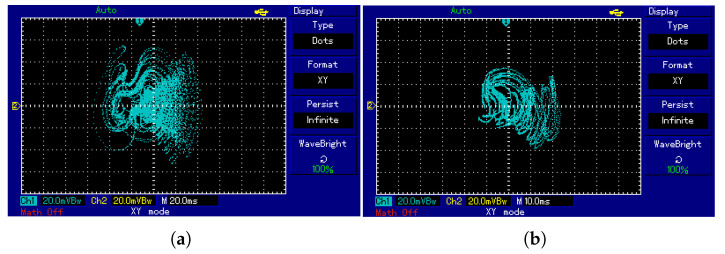
Phase portraits of system (2) based on DSP for different values of *m*: (**a**) m=2.5. (**b**) m=5.

Compared to analog circuits, digital circuits offer several advantages such as high precision, programmability, and low fault tolerance. DSPs are equipped with powerful data processing capabilities, are cost-effective, and are widely applied in engineering practice. Therefore, in this section, the hardware implementation of system (2) is carried out using a DSP chip. The overall hardware platform for the system implementation is shown in Figure 19. An independent emulator, XDS100V1, is used for program development and simulation. Data processing is handled by the TMS320F28335 chip, and the hardware board integrates a Digital-to-Analog Converter module, which converts the digitally generated signals into analog signals. Finally, the analog signals output by the DAC are sent to an oscilloscope for display. The system (2) is decomposed using Euler’s method. On the PC side, the development environment Code Composer Studio (CCS) is used, and the program is written in the C language and loaded onto the DSP chip. The parameters and initial conditions are set the same as those in Figure 4a,b, with the iteration step size set to h=0.001. To better display the simulation results, the oscilloscope image is represented using dots. The phase portrait is shown in Figure 20. The simulation results are consistent with the MATLAB simulation results in Figure 4a,b as well as the analog circuit simulation results in Figure 16, indicating that the system can be successfully implemented on the DSP platform.

## 5. Synchronization Control and Data Encryption Transmission

To fully leverage the advantages of system offset control, this paper implements synchronous control for a system incorporating three offset constants. Building upon this synchronous control, it further enables secure communication of binary digital information.

### 5.1. Synchronization Control

The system (11) is rewritten as shown in Equation (16), serving as the drive system.

(16) $$\left\{\begin{array}{c} {\dot{x}}_{1}=x_{2}+p_{2}-\left(x_{1}+p_{1}\right)+\left(x_{2}+p_{2}\right)\left(x_{3}+p_{3}\right)+m\left|x_{3}+p_{3}\right|-n\tanh\left(x_{3}+p_{3}\right)\\ {\dot{x}}_{2}=a\left(x_{1}+p_{1}\right)+b\left(x_{2}+p_{2}\right)-b\left(x_{1}+p_{1}\right)\left(x_{3}+p_{3}\right)\\ {\dot{x}}_{3}=\left(x_{1}+p_{1}\right)\left(x_{2}+p_{2}\right)-c\left(x_{3}+p_{3}\right) \end{array}\right.$$

where a=0.6, b=2, c=3, m=2, n=6, and the initial values of the system are (1, 2, 1). The system (11) is rewritten, and a controller is added, as shown in Equation (17), serving as the response system.

(17) $$\left\{\begin{array}{c} {\dot{y}}_{1}=y_{2}+p_{2}-\left(y_{1}+p_{1}\right)+\left(y_{2}+p_{2}\right)\left(y_{3}+p_{3}\right)+m\left|y_{3}+p_{3}\right|-n\tanh\left(y_{3}+p_{3}\right)+u_{1}\\ {\dot{y}}_{2}=a\left(y_{1}+p_{1}\right)+b\left(y_{2}+p_{2}\right)-b\left(y_{1}+p_{1}\right)\left(y_{3}+p_{3}\right)+u_{2}\\ {\dot{y}}_{3}=\left(y_{1}+p_{1}\right)\left(y_{2}+p_{2}\right)-c\left(y_{3}+p_{3}\right)+u_{3} \end{array}\right.$$

where a=0.6, b=2, c=3, m=2, n=6, and $u_{i}\left(i=1,\;2,\;3\right)$ represents the controller. The initial values of the system are (1, 1, 1). The error system is defined as

(18) $$\left\{\begin{array}{c} e_{1}=y_{1}-x_{1}\\ e_{2}=y_{2}-x_{2}\\ e_{3}=y_{3}-x_{3} \end{array}\right.$$

From Equations (16) to (17), we obtain

(19) $$\left\{\begin{array}{c} {\dot{e}}_{1}=-e_{1}+\left(p_{3}+1\right)e_{2}+p_{2}e_{3}+f_{1}+u_{1}\\ {\dot{e}}_{2}=\left(a-bp_{3}\right)e_{1}+be_{2}-bp_{1}e_{3}+f_{2}+u_{2}\\ {\dot{e}}_{3}=-ce_{3}+p_{2}e_{1}+p_{1}e_{2}+f_{3}+u_{3} \end{array}\right.$$

where

$$\begin{array}{c} f_{1}=y_{2}y_{3}-x_{2}x_{3}+m\left(\left|y_{3}+p_{3}\right|-\left|x_{3}+p_{3}\right|\right)+n\left(\tanh\left(x_{3}+p_{3}\right)-\tanh\left(y_{3}+p_{3}\right)\right)\\ f_{2}=bx_{1}x_{3}-by_{1}y_{3}\\ f_{3}=y_{1}y_{2}-x_{1}x_{2} \end{array}$$

To achieve synchronization control of the system, the controller is designed as

(20) $$\left\{\begin{array}{c} u_{1}=-f_{1}\\ u_{2}=-f_{2}-\left(b+1\right)e_{2}\\ u_{3}=-f_{3} \end{array}\right.$$

From Equation (20), we obtain

(21) $$\left\{\begin{array}{c} {\dot{e}}_{1}=-e_{1}+\left(p_{3}+1\right)e_{2}+p_{2}e_{3}\\ {\dot{e}}_{2}=\left(a-bp_{3}\right)e_{1}-e_{2}-bp_{1}e_{3}\\ {\dot{e}}_{3}=-ce_{3}+p_{2}e_{1}+p_{1}e_{2} \end{array}\right.$$

For Equation (21), the Lyapunov function is constructed as shown in Equation (22).

(22) $$V=\frac{1}{2}e_{i}^{T}e_{i},\;\;\;\;\;\;\;i=1,2,3$$

The derivative of the Lyapunov function is obtained as

(23) $$\begin{array}{c} \dot{V}=e_{1}{\dot{e}}_{1}+e_{2}{\dot{e}}_{2}+e_{3}{\dot{e}}_{3}\\ =\left(p_{3}+1+a-bp_{3}\right)e_{1}e_{2}+2p_{2}e_{1}e_{3}+\left(p_{1}-bp_{1}\right)e_{2}e_{3}-e_{1}^{2}-e_{2}^{2}-e_{3}^{2} \end{array}$$

Let $p_{1}=0$, $p_{2}=0$, and $p_{3}=1.6$. Based on the system parameter values, it can be calculated that $\dot{V}=-e_{1}^{2}-e_{2}^{2}-e_{3}^{2}=e_{i}^{T}Pe_{i}<0$. Thus, the matrix *P* is negative definite, theoretically proving that synchronization control is achieved for systems (16) and (17).

**Figure 21:**
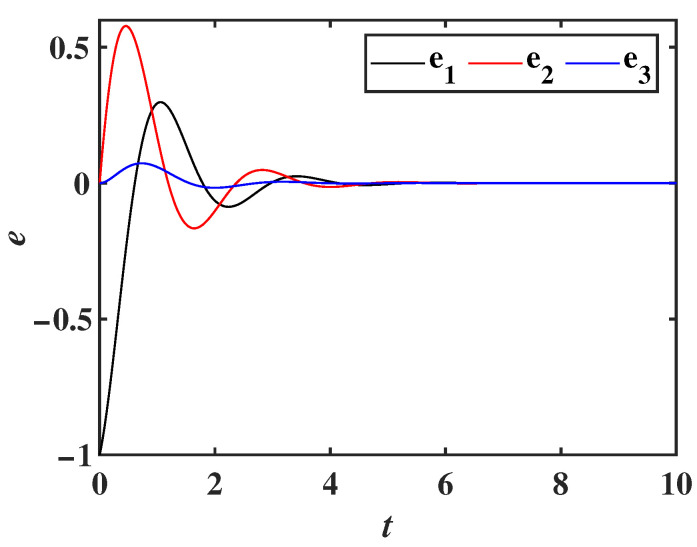
Error curve of the synchronization system.

To verify the effectiveness of the synchronization controller, numerical simulations are performed using MATLAB, and the error system curve is shown in Figure 21. As seen in the figure, the state variables of both the drive system and the response system achieve synchronization within 5 s, confirming the controllability of the new chaotic system and laying the foundation for secure communication in chaotic systems.

### 5.2. Data Encryption Transmission

**Figure 22:**
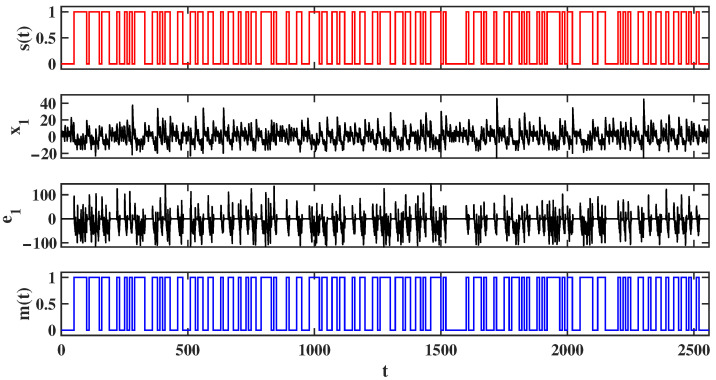
Digital information encrypted transmission process diagram.

In this section, synchronization control theory is applied to build a communication system using MATLAB for secure data transmission. A 256-bit random binary sequence was generated using the randi function. To facilitate a clear comparison between the encrypted and decrypted data, each 1-bit binary data was extended to a duration of 10 *s*. The three offset constants in the drive system (16) are modulated as follows: $p_{1}=s\left(t\right)\times offset$, $p_{2}=s\left(t\right)\times offset$ and $p_{3}=1.6+s\left(t\right)\times offset$, where offset is a constant. In the previous analysis, the offset was shown to have a relatively wide range, implying that the data encryption and transmission system possesses a large key space. In the current simulation, we choose offset=10. The offset constants for system (17) are set as $p_{1}=0$, $p_{2}=0$ and $p_{3}=1.6$. When the transmitter sends a binary 0, the offset values of the drive system and the response system are the same, achieving synchronization, and thus the error system is zero. When the transmitter sends a binary 1, the offset values of the drive system and the response system are different, and synchronization is not achieved, meaning the error system is non-zero. At the receiver side, data decryption is performed based on the error signals, and the binary digital information is demodulated. The recovered information m(t) matches the transmitted information s(t), confirming the successful encrypted data transmission. The simulation results are shown in Figure 22. Here, s(t) represents the data sent by the transmitter, while $x_{1}$ is the modulated information in the drive system. The difference between $y_{1}$ in the response system and $x_{1}$ in the drive system is denoted as $e_{1}$, and m(t) is the decrypted information. By comparing the transmitted data s(t) with the received data m(t), it was found that the 256-bit binary sequence exhibited a bit error rate of 0 during transmission. It is worth noting that, due to the extension of the binary digits, the simulation duration was 2560 s, while the actual physical time was 0.406886 s, confirming the effectiveness and reliability of the encrypted data transmission.

## 6. Conclusions

This study successfully constructed a new chaotic system based on the classical Chen chaotic system by introducing an absolute value function, a hyperbolic tangent function, and a cross-product term into the first equation to enhance nonlinearity. In the new system, the system parameter *m* not only controls the system’s state but also generates three different types of chaotic attractors. MATLAB simulations confirmed that all variables in the system exhibit offset boosting behavior controlled by constants. By adjusting the offset constants of different variables, offset boosting in arbitrary single or multiple directions can be achieved. Furthermore, the simulations demonstrated that the offset in each direction has a broad range of possible values, and each offset constant can regulate the frequency of all system variables. This flexible control of offset and frequency provides a wide range of options for the engineering application of chaotic signals. By introducing control constants into the system variables, both the range of chaotic states and the system’s complexity were expanded. This simple operational approach offers convenience for increasing system complexity. Multisim circuit simulations further verified that the system exhibits different chaotic attractors with varying *m* values and confirmed the multi-directional offset boosting behavior. The DSP hardware platform also validated the physical realizability of the system. Finally, using the Chen chaotic system as the driving system and the newly constructed system as the response system, a synchronization controller was designed to achieve synchronization between the Chen chaotic system and the new system. Based on this, secure binary data transmission was accomplished, laying the foundation for secure communication applications in the new system. Although this study exploits the advantages of the system to achieve secure data transmission, it is currently limited to data transfer, and relevant evaluation metrics require further supplementation and optimization. Applying the constructed system to the efficient encryption and transmission of medical images will be a key focus of future research.

## Figures and Tables

**Table 1:** Offset boosting behavior and frequency control.

Reference	System Dimension	Multiple Chaotic Attractors	Offset Boosting	Frequency Control via Single Offset Constants
[9]	3	No	No	Controls single variable frequency
[18]	6	Yes	[0, 10]	No
[20]	3	No	[−10, 10]	Controls single variable frequency
[29]	3	No	$\left[-10^{9},\;10^{9}\right]$	Controls single variable frequency
[38]	3	No	[0, 10]	Controls all variable frequencies
This paper	3	Yes	$\left[-10^{9},\;10^{9}\right]$	Controls all variable frequencies

**Table 2:** LEs and phase diagrams for different $p_{1}$.

$p_{1}$	${\mathrm{LE}}_{1}$	${\mathrm{LE}}_{2}$	${\mathrm{LE}}_{3}$	Phase Diagram
$1\times 10^{8}$	0.3811	−0.023	−9.3309	Figure 7a
$-1\times 10^{8}$	0.3001	−0.032	−9.258	Figure 7b
$1\times 10^{9}$	0.4132	−0.092	−9.4216	Figure 7c
$-1\times 10^{9}$	0.2985	−0.083	−9.3621	Figure 7d

**Table 3:** Comparison of system dimensions and SE values from different references.

Reference	System Dimension	SE
[32]	3	a∈[0.1, 1], SE∈[0.26, 0.4]; d∈[4, 6], SE∈[0.1, 0.51]
[41]	3	a∈[0, 5], b∈[0, 5], c∈[0, 5], SE∈[0.2, 0.55]
[42]	3	a∈[5, 35], b∈[0, 35], SE∈[0.4, 0.65]
[43]	4	a∈[1.2, 1.6], SE∈[0.15, 0.52]; b∈[0, 0.4], SE∈[0.47, 0.54]; k∈[0.2, 2], SE∈[0, 0.5]
[33]	4	a∈[0, 6], b∈[0, 6], SE∈[0.15, 0.55]
This paper	3	a∈[1.1, 9.8], SE∈[0.6, 0.76]

## Data Availability

Data is contained within the article.
